# Gamma and beta oscillatory signatures of the human olfactory bulb encode odor valence and sampling

**DOI:** 10.1016/j.isci.2026.115126

**Published:** 2026-02-21

**Authors:** Shubin Li, Coralie Mignot, Susanne Weise, Thomas Hummel

**Affiliations:** 1 Department of Otorhinolaryngology, Smell and Taste Clinic, Technical University of Dresden, Fetscherstrasse 74, 01307 Dresden, Germany

**Keywords:** neuroscience, sensory neuroscience, cognitive neuroscience

## Abstract

As the first relay between peripheral and central olfactory systems, the human olfactory bulb (OB) plays a dynamic role in odor perception. This study investigated OB time-frequency responses to odors of different valences under active versus passive sampling. Thirty-two participants underwent either inhalation-synchronized (active) or respiration-independent (passive) odor stimulation while electrobulbogram signals were recorded. Across both sampling modes, OB activity followed a consistent sequence of early gamma, scattered beta bursts, and late gamma-beta oscillations. Active sampling induced stronger and more widespread gamma activity throughout processing. Participants were further grouped based on N1-P2 ERP amplitudes. ERP-sensitive individuals exhibited enhanced early low-beta OB activity, whereas ERP-insensitive individuals showed stronger mid-to-late gamma and beta responses, suggesting compensatory stimulus re-evaluation. These findings indicated that OB gamma and beta oscillations encoded odor valence while reflecting temporal processing differences shaped by sampling strategy and individual sensitivity, highlighting the flexibility of early-stage human olfactory processing.

## Highlights

• OB gamma and beta oscillations encoded odor valence during active and passive sampling
• Active sampling elicited stronger and more widespread OB gamma activity across time
• ERP-sensitive individuals showed early low-beta OB activity synchronized with PFC
• ERP-insensitive individuals showed stronger mid-to-late gamma and beta responses

## Graphical abstract

**Figure:**
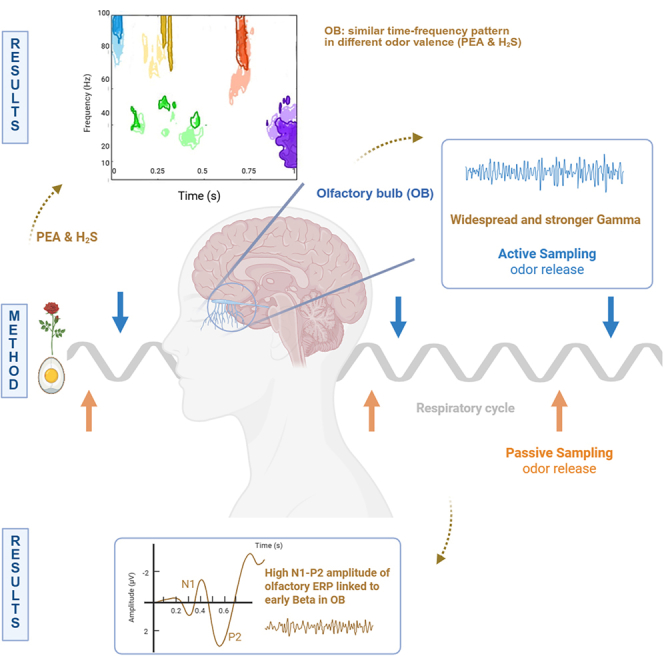

## Introduction

The olfactory bulb (OB), as the first and only relay station between the peripheral and central olfactory systems, plays a crucial role in human olfactory processing. It acquires olfactory signals from receptor neurons situated in the olfactory neuroepithelium^1^ and, after initial processing, transmits them to the primary olfactory cortex (piriform cortex, entorhinal cortex, and amygdala), and then to higher-order olfactory centers (orbitofrontal cortex, hippocampus, etc.).^2^ Therefore, the OB is likely involved in a wide range of human olfactory functions, including odor encoding, odor identification, and olfactory memory.^3^ However, our understanding of human OB and its functions remains limited. The best studied aspect is its volume. It changed with human aging, olfactory function,^4^^,^^5^^,^^6^ and certain conditions that may affect olfaction, e.g., head trauma, viral infections,^7^^,^^8^ neurodegenerative diseases,^9^^,^^10^ or psychiatric disorders.^11^

In contrast, many questions remained unexplored as for how the OB participated in human olfactory processing,^12^^,^^13^ e.g., the functional connectivity between OB activity and higher olfactory centers, the interaction between OB activity and human olfactory-related behavior, or whether OB activities can predict subsequent central processing. A new method has been developed using a reliable non-invasive technique for assessing OB activity in awake humans, known as the electrobulbogram (EBG).^14^ With this technique, it was found that odor valence was associated with gamma and beta frequencies of OB activity and individuals showed a faster response of unpleasant odors than to pleasant odors.^15^ Patients with idiopathic Parkinson’s disease showed specific EBG changes which were related to clinical features, such as medication, severity and duration of the disease.^16^ Furthermore, Nordén et al.^17^ found that OB communicated with piriform cortex mainly through gamma and beta bands to convey odor valence.

In chemosensory event-related potential (CSERP) research, stimulation is frequently designed to be dependent on the respiratory cycle,^18^^,^^19^ referred to as the active paradigm with odor delivery triggered by nasal inhalation. Inspiration played a crucial role in olfactory processing, as it primed the OB for incoming stimulation,^20^ influencing neural responses.^21^ This aligned with the “odor template” hypothesis, which suggested that the OB prepared for the next odor based on the sensory information from the previous one.^22^ Overall, these findings indicated that active and passive paradigms elicited distinct neural processing patterns in response to olfactory stimuli.

N1 and P2 components are classic components in CSERP research and their amplitudes exhibited sensitivity to changes in odor characteristics and quality, reflecting participants’ responsiveness to olfactory tasks and their sensitivity to odors. Previous research has shown that the CSERP amplitude N1 and P2 signals are influenced by various factors, such as age,^23^ gender,^24^ odor stimulus concentration,^25^ psychiatric disorders^26^^,^^27^^,^^28^ or alcoholism.^29^ As the first central structure to receive olfactory information, the OB processes first-hand exogenous characteristics of odors. The relationship between OB activity and CSERP components would provide valuable insights into how the OB processes odor characteristics and quality.

This study employed EBG technology to investigate OB activity during active and passive sniffing of odors with different valences, while also examining how individual sensitivity influenced these responses. By analyzing time-frequency dynamics within the 1-s post-stimulus interval, we aimed to identify the patterns of odor processing. These patterns would reveal both shared and condition-specific signatures of OB activity, highlighting how odor valence, sampling strategy, and individual differences in CSERP sensitivity shape olfactory processing.

## Results

### Descriptive statistics

Descriptive results of gender, age, and other demographic results were shown in Table 1.

**Table 1:** Descriptive statistics

	Passive	Active
Sex	9 men, 7 women	7 men, 9 women
Age (years)	25.8 ± 5.7	25.9 ± 4.6
Head circumference (cm)	57.0 ± 1.5	56.7 ± 1.7
Height (cm)	170.3 ± 9.9	173.6 ± 9.0
Weight (kg)	68.3 ± 13.1	67.5 ± 10.8

### Active and passive sampling

**Figure 1:**
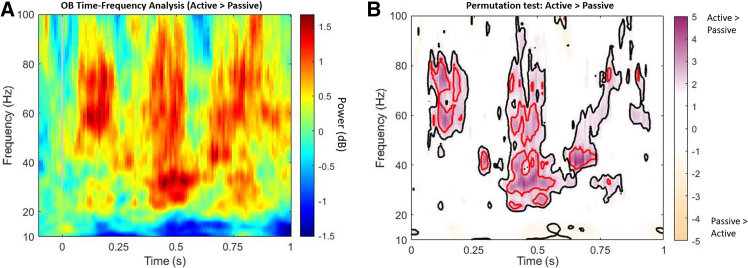
Time-frequency analysis and permutation test of OB between active and passive sampling The heatmaps depicted power and *t* values for the contrast “active sampling vs. passive sampling” groups, averaged across all odor valences. (A) Time-frequency power of the OB in the passive sampling group. Positive values indicated greater power in the active sampling group, while negative values indicated greater power in the passive sampling group. (B) Permutation test results for the power difference (active vs. passive). Regions with positive *t* values indicate significant effects where active > passive, whereas negative *t* values are significant effects where passive > active. Areas with *p* < 0.05 are outlined in black, and areas with *p* < 0.01 are outlined in red.

We found broader *ϒ*-band signals (∼30–100 Hz) of the “active sampling vs. passive sampling” contrast in a wide time range (early, 100–250 ms; middle, 300–500 ms; and late 600–800 ms; see Figure 1).

### Odor valence

**Figure 2:**
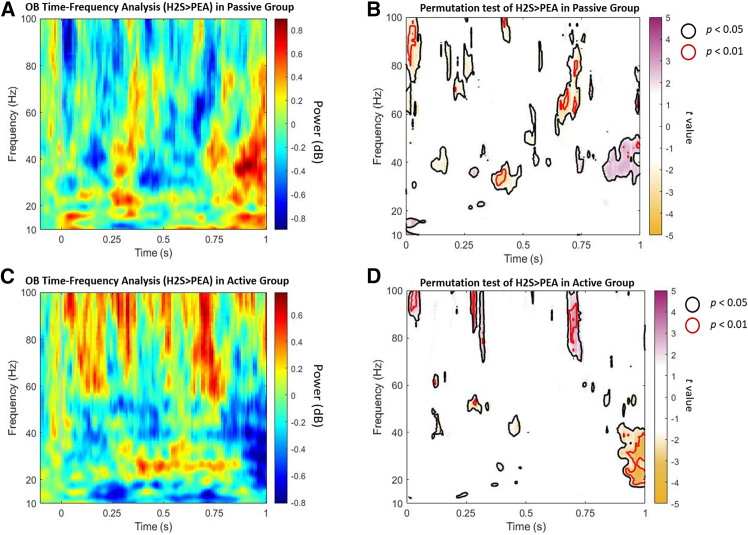
Time-frequency analysis of OB activities between H_2_S and PEA in active and passive sampling groups The heatmaps depicted power and *t* values for the contrast “H_2_S > PEA” groups. (A and C) showed the time-frequency power differences (H_2_S > PEA) of the OB in the passive (A) and active (C) sampling groups. (B and D) were the results of the corresponding permutation t-tests, with color representing *t* values for the H_2_S vs. PEA contrast. Areas with *p* < 0.05 are outlined in black, and areas with *p* < 0.01 are outlined in red. Regions with positive *t* values indicate significant effects where H_2_S > PEA, whereas negative *t* values are significant effects where PEA > H_2_S.

The OB signals in active and passive sampling showed in Figure 2. Distinct directional differences were observed between the two groups. In the significant oscillatory activities from 0 to 800 ms, H_2_S signals were stronger than PEA in the Active sampling group, whereas PEA signals were stronger than H_2_S in the Passive sampling group. This contrast reversed during the significant oscillations observed in the 800–1,000 ms window.

**Figure 3:**
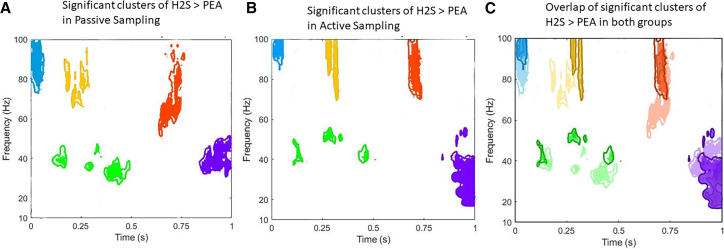
Significant time-frequency clusters of H_2_S > PEA in passive and active sampling (A) showed significant clusters from the permutation test contrasting H_2_S > PEA in the passive sampling group. (B) Significant clusters from the permutation test contrasting H_2_S > PEA in the active sampling group. (C) Overlap of significant clusters between the two groups, highlighting common and distinct regions of OB activity. The various colors only have a descriptive value to represent potential cluster similarities between the groups.

However, the comparison of both groups revealed similar activity patterns: from 0 to 100 ms, high gamma oscillations (70–100 Hz) were observed (blue areas in Figure 3); from 200 to 300 ms, another high gamma activity (60–100 Hz) emerged (yellow areas in Figure 3); during 100–500 ms, scattered low gamma clusters (30–50 Hz) were present (green areas in Figure 3); from 600 to 800 ms, middle to high gamma oscillations (50–100 Hz) reappeared (red areas in Figure 3); finally, in the 800–1,000 ms window, both low gamma and high beta (20–50 Hz) oscillations were detected (purple areas in Figure 3). Taken together, the main significant regions for the active and passive groups, along with their overlapping areas, are illustrated in Figure 3.

### OB activity and ERP sensitivity

**Figure 6:**
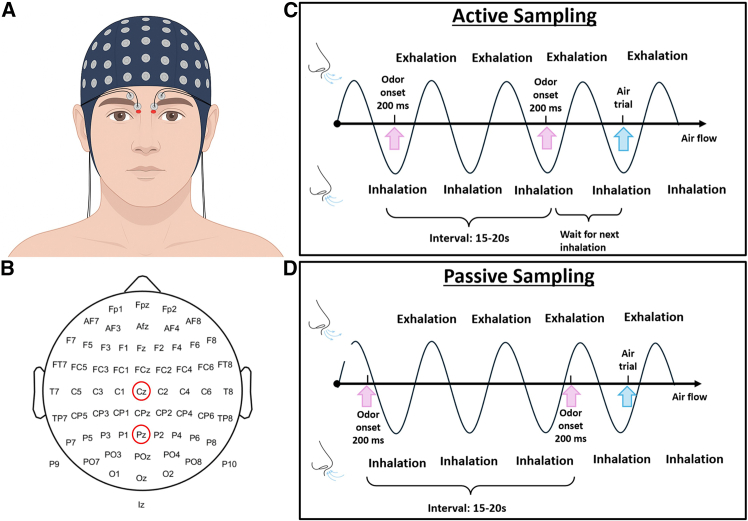
Overview of EBG electrode setup and task conditions (A) Electrode placement for EBG recording. Red areas indicated the approximate locations of OB. (B) The electrode positions of the 64-channel EEG system. The red circles indicated Pz and Cz. The right panel was experimental paradigms for odor presentation: (C) active sampling condition, in which odor delivery was triggered by the onset of inhalation; (D) passive sampling condition, where odors were delivered independent of the participant’s respiration.

Participants were ranked and grouped based on the N1-P2 amplitudes of the Pz and Cz electrodes (Figure 6B). Four participants with amplitudes falling within the middle 10% range (i.e., 45%–55%) were excluded. Those within the top 45% who responded stronger for ERP were classified as the “sensitive group” to odors, while those within the bottom 45% were categorized as the “insensitive group.” The sensitive group included three men and nine women, while the insensitive group comprised six men and six women (*Χ*^*2*^ *=* 1.6, *p* = 0.21).

**Figure 4:**
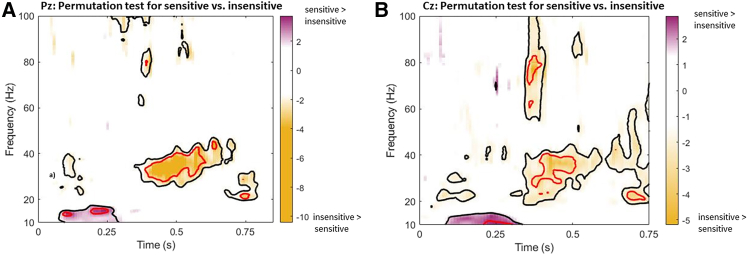
Permutation test of OB time-frequency analysis between sensitive and insensitive groups at Pz and Cz electrodes (A and B) show the *t* value maps for the contrast between sensitive and insensitive groups at the Pz and Cz electrodes, respectively. Areas with *p* < 0.05 are outlined in black, and areas with *p* < 0.01 are outlined in red. Regions with positive *t* values indicate significant effects where sensitive > insensitive groups, whereas negative *t* values denote significant effects where insensitive > sensitive.

At both the Pz and Cz electrodes, the sensitive and insensitive groups exhibited similar beta- and gamma-band oscillatory patterns. We observed 4 main significant areas. Compared to the insensitive group, participants in the sensitive group exhibited low beta oscillations (10–18 Hz) within the 100–300 ms time window. In contrast, the insensitive group demonstrated high gamma activity during 300–400 ms, and low gamma and high beta oscillations (around 30–45 Hz) enhanced in the 300–600 ms time window. Furthermore, a distinct 20 Hz oscillatory response showed during 600–750 ms (see Figure 4).

**Figure 5:**
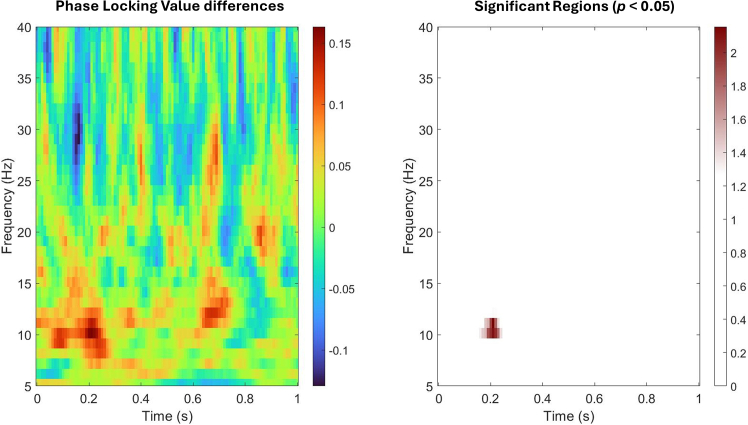
PLV differences and its permutation test of OB-PFC between sensitive and insensitive Groups (Left) The color scale represented group-level PLV differences between sensitive and Insensitive groups (Δ PLV). (Right) Significant regions revealed by non-parametric permutation test. Significance was visualized as −log10(p) values. Higher values indicated stronger significance with threshold corresponding to –log10 (0.05) ≈ 1.3.

We calculated the phase locking value (PLV) of OB and prefrontal cortex (PFC). The PLV differences of OB-PFC connectivity and its permutation test between sensitive and insensitive groups are shown in Figure 5. A significant effect emerged in the 10–12 Hz range around 200–300 ms, where sensitive participants exhibited stronger OB-PFC synchrony than insensitive participants.

## Discussion

The present study investigated the time-frequency characteristics of OB activity when perceiving odors of different valences and under different odor sampling conditions. Our findings showed that active sampling led to stronger and more widespread gamma oscillation across all phases. Odor with different valence evoked a consistent activation pattern under both active and passive odor administration, for both PEA and H_2_S: early gamma oscillations, scattered beta bursts from early to mid-phase, and late gamma followed by beta activity. Moreover, ERP-sensitive individuals exhibited early low-beta activity, whereas ERP-insensitive individuals showed enhanced mid-to-late gamma and beta responses, suggesting differing temporal dynamics in olfactory information processing.

This study conducted a time-frequency analysis of OB signals in both the active and passive sampling groups, comparing the differences between H_2_S and PEA conditions. Analyses also showed that in the early, middle, and late phases, active sampling exhibited broader gamma activation compared to passive sampling, reflecting more efficient γ-band transmission from OB to higher brain regions.^30^ This enhanced activation may result from the fact that, during active sampling, odor molecules are more efficiently delivered to the nasal cavity during inhalation, strengthening the transmission of odor information and leading to clearer and stronger neural signals in OB. Moreover, while our study focused on active versus passive odor sampling in humans, previous work has shown that different routes of odor access (orthonasal vs. retronasal) already lead to distinct temporal dynamics in the olfactory bulb of rodents.^31^ Together, these findings emphasized that input mode can strongly shape OB processing, even though the underlying manipulations differ.

Due to the difference in OB activity between active and passive sampling, we separately analyzed the OB responses to the two odors (H_2_S and PEA) in each sampling group. The results showed that the OB processing of odor valence exhibited both common and specific features between the active and passive sampling groups. Despite some temporal and frequency-related delays observed between the two groups, a similar activation pattern emerged consistently: 0–100 ms (80–100 Hz), 100–300 ms (70–100 Hz), 100–500 ms (scattered small clusters at 30–60 Hz), 600–750 ms (60–100 Hz), and 800–1,000 ms (15–50 Hz). Interestingly, H_2_S elicited stronger activation than PEA in the active sampling group, while the opposite was observed in the passive sampling group, where PEA showed greater activation. This phenomenon might be attributed to spontaneous respiratory modulation induced by different odor sampling modes.^32^ In the active odor presentation, odor release was synchronized with the participants’ inhalation, controlled by respiratory sensors that trigger odor delivery upon detecting the onset of breath. This design prevented participants from regulating their odor exposure. In contrast, in the passive sampling group, odor delivery was independent of participants’ breathing rhythm, allowing them to adjust their breathing depth and frequency when exposed to unpleasant odors like H_2_S. This spontaneous respiratory modulation potentially reduced the neural signals associated with negative olfactory stimuli, as participants subconsciously limit their exposure.^32^^,^^33^^,^^34^ No such behavioral adjustment was necessary for the slightly pleasant odor PEA, potentially explaining its heightened activation in the passive sampling group.^34^

These findings led us to observe a further odor valence processing pattern in human OB. The early high-gamma activation (0–100 ms) observed in our study was consistent with previous research, particularly in the Active Sampling group, where H_2_S exhibited significantly stronger oscillatory activity.^15^ This may reflect the human brain’s fast avoidance behavior toward hazardous stimuli.^15^^,^^35^ It is worth noting that, in the Passive Sampling group, PEA showed a stronger early high-gamma oscillation compared to H_2_S, indicating that early high-gamma activity is not exclusive to H_2_S but is also evident for PEA. Therefore, this may represent the OB’s initial physiological encoding of odor valence information: when the odor presentation is synchronized with breathing rhythm, the perception of negative odors is more distinct, potentially reflecting an unmodulated, more direct olfactory response. As indicated in previous literature, this can be considered as a rapid avoidance mechanism toward aversive odors.^15^ However, under more natural breathing conditions, this stronger gamma response was instead reflected in spontaneous respiratory adjustments to avoid negative odors.^35^^,^^36^

High-gamma activity in the OB remained significant between 200 and 300 ms, which still falls within an early processing stage. At the same time, extending to around 500 ms, we observed scattered small-cluster activations in the low-gamma and high-beta ranges in OB. In studies in rats tufted cells were thought to initiate early, fast-gamma, enabling rapid feature-specific transmission, whereas mitral cells contributed to later, slow-gamma that supported large-scale integrative binding,^37^^,^^38^^,^^39^ suggesting that EEG signals at different frequency bands may reflect the activity from different cell populations.

In our data, the gamma oscillations observed between 200 and 300 ms were likely to reflect the transmission and integration of olfactory valence information to central regions,^37^^,^^40^ such as the piriform cortex.^17^ The subsequent low-gamma and high-beta activations extending to 500 ms could represent cell-driven binding processes or feedback integration.^38^ Rather than representing different task-related oscillatory modes, gamma and beta appear to form a dynamic continuum of information processing. In other words, neural states can be dynamically modified depending on the task demands.^37^ The scattered activations pattern may indicate transient local computations as the system shifts between bottom-up sensory analysis and top-down modulation. However, this was derived from findings in rodents, and further investigation is required to substantiate and explore these possibilities.

In the late time window of 600–1,000 ms, we observed a sequential reactivation of gamma and beta oscillations. We speculated that this pattern may be related to the integration of valence information and the evaluation of odor hedonics. Previous research has demonstrated that although the actual hedonic assessment occurs in higher-order regions such as the orbitofrontal cortex (OFC) and anterior cingulate cortex (ACC), the OB served as the initial stage of olfactory sensory processing.^41^ Beta oscillations in the OB have been shown to be involved in hedonic integration and are functionally linked to reward and sensory-related processes in ACC, OFC, and the medial prefrontal cortex (mPFC).^41^

In addition, the result showed that during 800–1,000 ms, the direction of odor-related power differences was reversed compared to the early and mid-phases. This late inversion of power was consistently observed across participants, suggesting a secondary processing stage of odor information beyond the initial sensory response. However, the underlying remains unclear. One possible explanation could involve inhibitory rebound dynamics within the OB.^42^ As most EBG studies on odor valence so far focused on early activity within the first 250 ms,^15^^,^^17^ the present study is the first to explore odor valence processing on OB after 250 ms. If this pattern can be replicated in future studies, it should be further examined from multiple perspectives, including physiological and neuroimaging approaches.

To further explore how individual differences in ERP task sensitivity related to OB activity, we compared EBG signals between sensitive and insensitive participants. The sensitive group exhibited stronger low-beta oscillations (10–18 Hz) within 100–300 ms time window. PLV differences showed that ERP sensitive people’s OB and PFC had a significant synchronization in alpha and low beta waves. The phase synchronization between OB and PFC may reflect attentional or regulatory feedback, rather than direct sensory transmission. In other words, this alpha- and low beta-band synchrony likely represented top-down gating or modulation of olfactory input by the prefrontal cortex.^43^^,^^44^ Recent evidence also highlighted their role in predictive coding.^45^ The early emergence of beta in the sensitive group (100–300 ms) may thus reflect sensitive individuals may engage stronger cortical-bulbar interaction, which could help them process odor information more efficiently.^46^

Furthermore, the insensitive group exhibited significant low gamma oscillations (around 20–45 Hz, 70–80 Hz) within the 300–500 ms time window during olfactory processing, which we hypothesized could be a compensatory mechanism of its lack of early predictive coding. Frederick et al.^37^ reported a similar result that slow gamma (∼70 Hz) was stronger during more complex tasks. In the present study, the early processing deficits made ERP tasks more difficult to people in the insensitive group. Gamma oscillations are well-known for their role in feature binding in visual systems, for example encoding of features like color and shape.^47^^,^^48^ The 300–500 ms window is considered a mid-phase processing stage in sensory neuroscience, positioned between early feature encoding and late-stage decision-making. Therefore, the gamma oscillations in mid-phase could imply the OB’s attempts as a compensatory mechanism. Additionally, the 700–750 ms window might reflect a later stage of processing, when the brain is approaching the re-evaluation of sensory input. Therefore, the ∼20 Hz high-beta oscillations observed in the insensitive group during the late stage (∼750 ms) may reflect reprocessing of olfactory information. This re-evaluation likely remained part of the compensatory mechanisms in insensitive subjects, potentially involving processes such as error monitoring and adaptive adjustment of sensory signals, following the mid-phase period.

Overall, the consistent combination of gamma followed by beta oscillations may reflect a dynamic flow of information processing in OB. Based on our observation, gamma oscillation was widely associated with localized sensory processing, including feature binding and rapid encoding. In contrast, beta oscillations were more often linked to long-range communication, top-down prediction, integrative processing, and behavioral preparation.

For sampling strategy, active sampling showed stronger and broader gamma-band responses than passive sampling, indicating OB receives a stronger or more synchronized sensory input during active sampling. For different olfactory tasks, these two rhythms emerged at different time windows, representing sequential stages of OB involvement. In the early phase (∼50–300 ms), high-gamma activity likely reflected the initial processing of chemical and valence-related odor features, while low-beta power may represent early top-down preparatory influences from prefrontal areas that modulated OB and contributed to higher ERP sensitivity in some individuals. In the mid-latency range (∼300–700 ms), both odor valence and ERP sensitivity effects exhibited concurrent gamma and beta components. This may imply a transitional stage in which bottom-up odor encoding interacts with top-down feedback. Finally, in the late phase (after ∼750 ms), beta oscillations dominated both the odor valence and the ERP sensitivity effects, likely reflecting feedback from higher olfactory-related regions.

As mentioned above, rather than representing independent and fixed stages, this gamma-to-beta progression was more like a flexible continuum of neural processing, which could be dynamically shaped by task demands, respiratory cycle, or the odor valence.^30^^,^^37^^,^^49^ Such a continuum may enable the olfactory system to rapidly adapt its processing mode according to behavioral relevance or environmental uncertainty.

In conclusion, the present study focused on the time-frequency characteristics of OB when perceiving odors of different valences and under different odor sampling conditions. Human OB gamma and beta oscillations dynamically encoded odor valence in a consistent activation pattern under both active and passive odor administration, which could reflect its temporal dynamic processing, including initial encoding and integration. Moreover, active sampling led to stronger and more widespread gamma signals across all phases. Besides, OBs of ERP-sensitive individuals exhibited early low-beta activity, and it was synchronized with PFC, whereas ERP-insensitive individuals showed enhanced mid-to-late gamma and beta responses. This result could suggest that people sensitive to ERP tasks would actively predict and evaluate odor stimuli rather than passively receiving them, while insensitive people would exhibit more gamma and beta activities as a compensatory mechanism.

### Limitations of the study

Several limitations must be acknowledged in the present study. First, the source localization was performed using a standard head model rather than individualized head models. This approach may overlook the anatomical differences across participants. Future studies could benefit from the use of personalized head models to achieve more precise source localization.^50^ Second, the sample size in this study was relatively small. Increasing the sample size in future studies would enhance statistical power and generalizability of the findings. Third, participants’ respiratory frequency and amplitude were not directly recorded due to the limited function of the device. Including respiration measures in future experiments would provide a more accurate assessment of how breathing patterns relate to odor perception and neural responses.

## Resource availability

### Lead contact

Further information and requests for resources and information should be directed to and will be fulfilled by the lead contact, Shubin Li (shubin.li1@tu-dresden.de).

### Materials availability

The experiment consisted of two sessions, each presenting one of two odorants with clear supra-threshold intensity and opposite valence: the slightly pleasant phenylethyl alcohol (PEA, rose-like scent, 50%, v/v) and the unpleasant hydrogen sulfide (H_2_S, rotten-egg scent, ∼4 ppm).

### Data and code availability

• The raw data supporting the findings of this study are available in Mendeley Data: https://doi.org/10.17632/8b2wc5sx2f.1.
• The MATLAB analysis scripts used in this study are available in Mendeley Data: https://doi.org/10.17632/8b2wc5sx2f.1.
• Supporting files required for analysis (e.g., electrode layout) are available in Mendeley Data: https://doi.org/10.17632/8b2wc5sx2f.1.

## Acknowledgments

We would like to express our gratitude to Frans Norden and Johan Lündstrom for their generous guidance and support.

Funding: This research was supported by 10.13039/501100001663Volkswagenstiftung (project PERCEPTRONICS, Az 9B396). Open Access funding enabled and organized by Projekt DEAL.

## Author contributions

Conceptualization, S.L., C.M., and T.H.; data curation, S.L.; formal analysis, S.L.; funding acquisition, T.H.; investigation, S.L. and C.M.; methodology, S.L. and C.M.; project administration, T.H.; supervision, T.H.; visualization, S.L.; data interpretation, S.L., C.M., S.W., and T.H.; writing – original draft, S.L.; writing – review and editing, S.L., C.M., S.W., and T.H.

## Declaration of interests

All authors declare no competing interests.

## STAR★Methods

### Key resources table

**Table:** 

REAGENT or RESOURCE	SOURCE	IDENTIFIER
**Chemicals, peptides, and recombinant proteins**		

Phenylethyl alcohol (PEA)	Sigma-Aldrich	CAS: 60-12-8
Hydrogen sulfide (H_2_S)	Air Liquide	CAS: 7783-06-4

**Deposited data**		

Raw EEG and EBG data	This paper	https://doi.org/10.17632/8b2wc5sx2f.1
Supporting files required for analysis (e.g., electrode layout)	This paper	https://doi.org/10.17632/8b2wc5sx2f.1

**Software and algorithms**		

MATLAB 2024	MathWorks	RRID:SCR_001622
FieldTrip toolbox 2024	Oostenveld et al.	RRID:SCR_004849; https://github.com/fieldtrip/fieldtrip/releases
MATLAB analysis scripts	This paper	https://doi.org/10.17632/8b2wc5sx2f.1

**Other**		

Computer-controlled olfactometer	Burghart	N/A
Breathing sensor	Burghart	N/A

### Experimental model and study participant details

#### Participants

In a pilot study, 20 participants (9 men, 11 women; mean age = 28.8 ± 4.4 years) were asked to evaluate the intensity and pleasantness of odors used in the experiments. In the formal study, we recruited 32 healthy participants (16 women: age = 25.9 ± 6.0 years; 16 men: age = 25.8 ± 4.2 years; details see Table 1). All participants provided written informed consent and completed a medical history questionnaire prior to the experiment. They reported themselves to be non-smoking, never had nasal surgeries, had normal olfactory function, never had head trauma, or neurological/psychiatric disorders. The 16-item odor identification of the Sniffin’ Sticks test^51^ was conducted to ascertain normal olfactory function, and people who scored under 12 were excluded. Given the effect of metabolic state on human perception of non-food odors,^52^ participants were asked to fast for at least 2 h before the formal experiment. This restriction ensured that all participants were not in a sated state to maximize their sensitivity to the odors. One participant was excluded due to a low odor identification score, and 2 participants were excluded due to technical issues with the EEG recordings, leaving a total of 32 participants. Details of participants were reported in results.

The study was conducted according to the Declaration of Helsinki at the Smell and Taste Clinic at the Department of Otolaryngology at the University Clinic Carl Gustav Carus Dresden (Germany) and approved by the local ethics board (EK-400082021_1).

### Method details

#### Odor stimuli and procedure

The EEG experiment took place in an air-conditioned ventilated room. Odors and their concentration were selected based on previous olfactory research.^53^^,^^54^^,^^55^^,^^56^^,^^57^ In order to prevent nasal mucosa irritation, odors were presented in a constant airflow of controlled temperature and humidity, administered by a computer-controlled olfactometer (Burghart, Holm, Germany), with a temperature of 37°C, a relative humidity of 80%, and a total airflow of 8 L/min). During the experiment, participants were asked to listen to white noise with headphones (approximately 50 dB pressure level) and played a simple tracking task on computer^58^ to help them focus/stabilize attention and reduce external distractions.

Each session comprised 80 trials, 40 odor stimuli and 40 odorless trials of 200 ms duration. Participants were instructed to breathe naturally to remain as still and relaxed as possible to minimize movement-related EEG artifacts throughout the experiment. To prevent habituation, odor and air trials alternated throughout the session. Sixteen participants underwent an active sampling design, in which odor delivery was synchronized with inhalation, while the other 16 participants followed a passive sampling design, where odor delivery was not synchronized to breathing. In the passive sampling group, odors were presented automatically every 15–20 s in a pseudo-randomized order. The scents were directly introduced to the participants’ nostrils, allowing them to perceive the odors even without actively inhaling (Figure 6). In the active sampling group, odors were time-locked to participants’ nasal inhalation using approximately the same pseudo-randomized interstimulus interval as in the passive sampling group (Figure 6). A breathing sensor (ref. OL027, LA-07-04005, Burghart, Holm, Germany) coupled to the olfactometer was used to detect nasal inhalation and initiate a transistor–transistor logic (TTL)-compatible pulse signal, thus precisely synchronizing odor delivery with breathing events. In active sampling group, the onset of each stimulus was triggered by the participant’s next inhalation, as detected by the breathing sensor. This approach ensured that the duration of the sessions was approximately equal for both groups (16–18 min, with a minimum 3-min break between sessions).

#### Electrodes placement and preprocessing

Sixty-four scalp EEG electrodes were placed according to the international 10/20 system, accompanied by 4 external EBG electrodes positioned on the forehead (Figure 6). Signal acquisition was conducted at a sampling rate of 512 Hz (Biosemi, Amsterdam, NL). Data from both EEG and EBG electrodes were incorporated to interpolate surface potentials across the scalp and forehead. Before the experiment started, electrode offsets were manually checked and adjusted as needed to ensure they remained below the predefined threshold of 30 mV.

EEG data preprocessing was conducted using FieldTrip toolbox 2024.^59^ The zero point was defined as the onset of odor delivery. The continuous data were then segmented into epochs from −500 to 2000 ms relative to stimulus onset. Baseline correction was applied to each trial using the −200 to 0 ms pre-stimulus interval relative to odor onset. A high-pass filter (2 Hz), low-pass filter (200 Hz), and notch filter (50 Hz) were applied to remove slow drifts, high-frequency noise, and power line interference. Bad channels were detected based on variance measures and visual inspection, subsequently interpolated using spline interpolation, and the data were re-referenced to the average reference. Automatic artifact rejection was then performed based on predefined thresholds, with jump artifacts identified using a cutoff of 80 μV and muscle artifacts filtered in the 110–140 Hz range with a threshold of 12 μV. Independent component analysis (ICA) was employed to identify and remove artifacts related to ocular and muscle activity. This preprocessing pipeline was applied consistently across all participants to ensure data quality and comparability for subsequent analyses.

#### Source reconstruction

We performed source reconstruction to confirm that the signal sources correspond to the OB region. It was conducted by using the FieldTrip toolbox within MATLAB 2024b. Preprocessed EEG data, following ICA-based artifact correction, were analyzed separately for each subject and condition. Source modeling was based on a standard head model and an MNI-aligned template MRI. A standard 10-mm MNI source grid was aligned with the template MRI to define the source space. We employed the eLORETA algorithm for distributed source reconstruction. The forward model was computed using the three-shell boundary element method (BEM), and EEG sensor positions were aligned to a standard 10–20 electrode layout. Our analysis focused on two predefined regions of interest (ROIs), corresponding to the left and right OB. OB coordinates were defined in MNI space based on previous neuroimaging studies: left OB at (−4, 40, −30) and right OB at (4, 40, −30). Source activity was estimated for each trial-averaged dataset and projected onto the source grid.

### Quantification and statistical analysis

Data for each subject and condition were loaded and preprocessed, including Independent Component Analysis (ICA) to remove artifacts and enhance signal quality. Time-frequency decomposition was performed using multitaper spectral estimation (10–100 Hz) on selected EEG channels with trial windows defined from −0.1s to 1s. Trials exhibiting high variance, as determined by z-scores, were excluded.

Grand averages were calculated for each condition and group, reflecting the mean time-frequency representation of neural oscillations across trials. Following this, baseline correction was applied using a [−0.2s–0s] window relative to stimulus onset. This time window was chosen to capture pre-stimulus neural activity, ensuring that subsequent analyses reflect relative changes from a consistent reference point. The baseline-corrected data were then converted to decibel (dB) changes for normalization across conditions. Next, condition-specific contrasts were computed by subtracting the time-frequency representations of the air condition from the odor condition within each group.

To quantify ERP sensitivity, we extracted the N1 and P2 amplitudes at Pz and Cz electrodes for each participant, as previous research has identified them as the most reliable and sensitive for olfactory ERPs, showing maximal N1–P2 amplitudes and strong responsiveness to valence-related late components.^14^^,^^60^^,^^61^^,^^62^^,^^63^ We manually inspected the N1 and P2 amplitudes for all participants. Participants whose N1-P2 amplitudes fell within the middle 10% range were excluded. The remaining participants were then divided into two groups based on amplitude distribution: the top 45% were classified as sensitive, while the bottom 45% were classified as insensitive. The N1-P2 amplitudes at Pz and Cz both showed significant differences (Pz: sensitive = 4.73 ± 0.82, insensitive = 3.04 ± 0.61, *t* = 7.35, *p* < 0.001; Cz: sensitive = 5.57 ± 1.91, insensitive = 3.18 ± 0.66, *t* = 5.81, *p* < 0.001).

Group-level comparisons were subsequently performed by contrasting summed differences between the conditions (H_2_S vs. PEA in each sampling design, active vs. passive sampling groups, sensitive vs. insensitive groups). Non-parametric permutation tests based on the Monte Carlo method were applied separately for all contrasts: Active vs. Passive, H_2_S vs. PEA in each sampling group, and Sensitive vs. Insensitive. For each contrast, condition labels were randomly shuffled across 1000 iterations.

Based on the ERP task sensitivity grouping, we performed a Phase Locking Value (PLV) analysis to investigate the consistency of phase synchronization between the OB (4 OB electrodes) and prefrontal cortex (PFC electrodes: Fz, F3, F4, AF3, AF4, Fp1, Fp2) during olfactory processing. Within each region, signals were averaged to obtain one representative time series for OB and one for PFC. Time–frequency decomposition was performed using Morlet wavelets (width = 5) over a frequency range of 0–100 Hz and a time resolution of 10 ms, implemented in FieldTrip 2024. The resulting complex values were used to calculate the PLV between OB and PFC, showing how their phases were synchronized over time and frequency for each participant. Group-level PLV maps were averaged within each sensitivity group, and the differences between the sensitive and insensitive groups were computed at each time-frequency point. To assess statistical significance, a non-parametric permutation test (1,000 randomizations) was applied to generate empirical *p*-values, which were visualized as –log10(p) values to improve contrast. All figures generated by MATLAB2024b.
